# Perfusion indices revisited

**DOI:** 10.1186/s40560-017-0220-5

**Published:** 2017-03-14

**Authors:** Ahmed Hasanin, Ahmed Mukhtar, Heba Nassar

**Affiliations:** 1 Anesthesia and Critical Care Medicine, Cairo University, Giza, Egypt; 2 Critical Care Department, El-Ameen Hospital, Taif, Kingdom of Saudi Arabia

**Keywords:** Perfusion indices, Lactate, Shock

## Abstract

Monitoring of tissue perfusion is an essential step in the management of acute circulatory failure. The presence of cellular dysfunction has been a basic component of shock definition even in the absence of hypotension. Monitoring of tissue perfusion includes biomarkers of global tissue perfusion and measures for assessment of perfusion in non-vital organs.

The presence of poor tissue perfusion in a shocked patient is usually associated with worse outcome. Persistently impaired perfusion despite adequate resuscitation is also associated with worse outcome. Thus, normalization of some perfusion indices has become one of the resuscitation targets in patients with septic shock.

Although the collective evidence shows the clear relation between impaired peripheral perfusion and mortality, the use of different perfusion indices as a resuscitation target needs more research.

## Background

Monitoring of tissue perfusion is an essential step in the management of acute circulatory failure. The presence of cellular dysfunction has been a basic component of shock definition even in the absence of hypotension [1]. The ideal parameter for tissue perfusion should be rapid, non-invasive, and easily measured without the need of advanced skills. Evaluation of tissue perfusion includes clinical evaluation as well as biomarkers. Popular biomarkers of tissue perfusion such as serum lactate and central venous oxygen saturation are indicators of global tissue perfusion. Monitoring of peripheral circulation especially in non-vital organs added new insights for monitoring of tissue perfusion. Peripheral non-vital organ perfusion deteriorates earlier and improves later than vital organs. Assessment of peripheral circulation has become easier after introduction of new non-invasive devices as well as clinical scoring systems.

In this article, we provide a comprehensive updated review for all the available indices for monitoring of tissue perfusion. The advantages and disadvantages of each method will be mentioned. Thoughts for future research and gaps in literature will also be highlighted (Table 1).

**Table 1:** Perfusion indices: evidence and disadvantages

Measure	Evidence	Disadvantages
Lactate	Meta-analysis	Needs blood sampling Laboratory time Could be elevated in other conditions than shock
ScvO_2_	RCT	Needs central catheter Less useful under anesthesia
P (v-a) CO_2_	Cohort	Needs central catheter Needs blood sampling Laboratory time
Temperature gradients	Cohort	*T*_c-toe_ is affected by hypothermia and room temperature
LV strain	Cohort	Needs expert operator
Skin mottling	Cohort	Limited value in burns, amputations, and in dark skin
Capillary refill time	Cohort	Index CRT: inter-rater variability Knee CRT: limited value in dark skin
PPI	Cohort	Needs an expensive device and electrodes
StO_2_	Cohort	Needs NRIS and electrodes Less accurate in septic shock Less accurate in early shock stages
Ptco_2_	Cohort	Needs special monitor and electrodes No definitive cutoff value
OCT	RCT	Needs special monitor and electrodes Restricted to intubated patients
Sublingual microcirculation	Cohort	Needs special device

*CRT* capillary refill time, *LV* left ventricular, *NRIS* near-infrared spectroscopy, *OCT* oxygen challenge test, *Ptco*_*2*_ subcutaneous partial oxygen pressure, *PPI* peripheral perfusion index, *P (v-a) CO*_*2*_ central-venous-arterial oxygen gradient, *RCT* randomized controlled trial, *ScvO*_*2*_ central venous oxygen saturation, *StO*_*2*_ tissue oxygen saturation, *T*_*c-toe*_ central-to-toe temperature gradient, *T*_*skin-diff*_ temperature gradient between fingertip and forearm

## Cardiac output as a determinant of tissue perfusion

The balance between oxygen delivery (DO_2_) and oxygen consumption (VO_2_) considered the mainstay of understanding the concept of tissue perfusion and the development of organ dysfunction. In a steady state, the VO_2_ constitutes only 25% of DO_2_. In a shock state, the VO_2_ increased out of proportion of DO_2_ to the point that DO_2_ falls below a critical threshold where the VO_2_ is dependent on DO_2_. Below that point, organ perfusion will be critically impaired and transition to anerobic metabolism will occur [2].

Because the main determinant of DO_2_ is the cardiac output (CO), most patients’ evaluation and resuscitation strategies depend on evaluating and optimizing this parameter. Assessing CO is performed by invasive, minimally invasive, and non-invasive methods [3]. More recently, surviving sepsis campaign recommended the routine use of echocardiography during initial assessment of patients with septic shock [4].

## Markers of global perfusion

### Lactate

#### Background

Lactate is the end product of anerobic glycolysis [5]. Serum lactate level increases in states of cellular hypoxia or low peripheral perfusion; thus, serum lactate level is considered a surrogate of cellular perfusion [5].

#### Selectivity and sensitivity in clinical settings

Lactate is the most frequently used marker of tissue perfusion [6]. Lactic acidosis is a predictor of in-hospital mortality in septic shock [7, 8]. Increased lactate clearance during resuscitation of septic shock was associated with improved outcomes [9].

#### Usefulness in other settings

Lactic acidosis is a predictor of in-hospital mortality in trauma [10] and cardiac arrest [11].

#### Degree of invasiveness

It is minimally invasive but needs available blood gas analysis.

### Mixed and central venous oxygen saturation

#### Background

Mixed venous oxygen saturation (SvO_2_) is an indicator for adequate oxygen delivery (DO_2_) [1]; thus, the change in SvO_2_ reflects the change in cardiac output as long as other determinants of DO_2_ are stable [1]. In healthy individuals, SvO_2_ value is 75% while in critically ill patients, its value is 70% [1]. Central venous oxygen saturation (ScvO_2_) is another indicator for oxygen delivery; however, the evidence on the correlation between ScvO_2_ and SvO_2_ is not clear [12, 13]. ScvO_2_ reflects perfusion status in the upper body and is not affected by blood coming from the lower body nor coronary sinus [14]. In healthy individuals, ScvO_2_ is lower than SvO_2_ [15]; however, in shocked patients, ScvO_2_ may exceed SvO_2_ by up to 20% due to redistribution of blood to the upper body [16, 17].

#### Selectivity and sensitivity in clinical settings

Although ScvO_2_ and SvO_2_ are not interchangeable, ScvO_2_ is still useful as a predictor of outcome in septic shock. Persistently low ScvO_2_ during resuscitation is associated with poor outcomes in septic shock patients [18, 19]. Despite its clinical value, maintenance of ScvO_2_ is no longer a resuscitation goal in the new surviving sepsis campaign guidelines [4].

#### Usefulness in other settings

Despite the limited value of ScvO_2_ under general anesthesia, it was reported as a valuable measure in cardiac surgery patients where lower ScvO_2_ values were associated with more complications [20]; moreover, in a randomized controlled trial, maintenance of ScvO_2_ of at least 70% was associated with lower complications in cardiac surgery patients [21].

#### Limitations and degree of invasiveness

In addition to the need to central venous catheter, ScvO_2_ is usually less informative under general anesthesia because the use of neuromuscular blockers decreases oxygen consumption; moreover, the use of high fraction of inspired oxygen usually (FiO_2_) corrects hypoxemia and increases DO_2_ [22].

### CO_2_ gap (P (v-a) CO_2_)

#### Background

The difference between PCO_2_ in central venous blood and PCO_2_ in arterial blood is known as central-venous-arterial CO_2_ gap (P (v-a) CO_2_). P (v-a) CO_2_ has been considered as an indicator of the adequacy of venous blood flow to wash out CO_2_ in peripheral tissues [23]. Elevated P (v-a) CO_2_ (above 6 mmHg) occurs in cases of decreased systemic blood flow. Normalization of P (v-a) CO_2_ during resuscitation was associated with normalization of serum lactate [24].

#### Selectivity and sensitivity in clinical settings

P (v-a) CO_2_ is negatively correlated with cardiac output in septic shock patients [25]; thus, P (v-a) CO_2_ is a useful parameter to assess the adequacy of tissue perfusion during resuscitation of patients in septic shock [24]. Persistence of high P (v-a) CO_2_ during early resuscitation of septic shock is associated with poor outcomes [26].

In cases with decreased ScvO_2_, a high P (v-a) CO_2_ denotes that tissue hypoperfusion is due to inadequate cardiac output, whereas normal P (v-a) CO_2_ denotes that improving cardiac output is unlikely to improve the oxygen delivery to the tissues [23]. In cases of normal ScvO_2_ and P (v-a) CO_2_ with evidence of anerobic metabolism (i.e., elevated serum lactate), manipulation of macrocirculation would be not effective to improve the condition because this denotes mitochondrial or microcirculatory disturbances [23].

#### Usefulness in other settings

In addition to its value in septic shock patients, P (v-a) CO_2_ is also negatively correlated with cardiac output in cardiogenic shock patients [27] and patients with severe isovolemic anemia [28].

#### Limitations and degree of invasiveness

Assessment of P (v-a) CO_2_ needs the presence of both arterial and central venous lines.

### Left ventricular strain

#### Background

Left ventricular longitudinal strain (LVS) is defined as “the percentage distance shortening of the endocardium along its length” [29]. The traditional parameter for assessment of left ventricular function is ejection fraction; however, this parameter could be confounded by cardiac preload and heart rate; moreover, it is highly operator dependent; thus, LVS appears as a novel method for evaluation of systolic function that could help in early detection of myocardial ischemia [30].

#### Selectivity and sensitivity in clinical settings

In a prospective observational study [31], the ability of LVS as a measure for oxygen delivery was evaluated. LVS was associated with elevated serum lactate and low ScvO_2_ in patients with septic shock. The findings of the aforementioned study suggest that LVS could be a non-invasive surrogate for assessment of tissue perfusion.

#### Usefulness in other settings

There is no available evidence.

#### Limitations and degree of invasiveness

This parameter is limited by the need of an expert echocardiography operator [31]. A summary for global and local indices is provided in Table 1.

## Markers of local perfusion

### Temperature values and gradients

#### Background

Skin temperature is a traditional sign of peripheral vasoconstriction. Critically ill patients with cold skin temperature have lower cardiac index, lower Svo2, and higher serum lactate compared to patients with warm skin temperature [32]. Temperature gradients are better subjective methods for assessment of peripheral perfusion [33, 34].

Central-to-toe temperature (*T*_c-toe_) is the difference between central temperature measured at the tympanic membrane and temperature at the ventral surface of the big toe measured by a skin probe. *T*_c-toe_ gradient has been used as a measure of peripheral vasoconstriction; however, it has the disadvantage of being affected hypothermia as well as room temperature [34].

The temperature gradient between fingertip and forearm (*T*_skin-diff_) has also been reported as a marker of peripheral vasoconstriction. *T*_skin-diff_ is the difference between the temperatures at the index and at the radial side of the forearm measured using two skin probes. *T*_skin-diff_ has the advantage of not being affected by the ambient temperature because the change in ambient temperature affects both fingertip and forearm equally [34, 35].

#### Selectivity and sensitivity in clinical settings

*T*_c-toe_ more than 7 °C and *T*_skin-diff_ more than 0 °C are indicators for vasoconstriction [34]. *T*_skin-diff_ more than 2 and 4 °C indicates the presence of intermediate and severe vasoconstriction respectively [35]. There is an association between *T*_skin-diff_ and poor outcomes in abdominal surgery patients [35] and in patients with acute circulatory failure.

#### Usefulness in other settings

The persistence of peripheral perfusion alterations after reversal of therapeutic hypothermia are associated with poor outcomes in cases of out-of-hospital arrest [36]. In cardiac surgery patients, the transition from peripheral vasoconstriction to vasodilatation (measured by skin surface gradient) is associated with shorter time to extubation after both normothermic and hypothermic cardiopulmonary bypass [37].

#### Limitations and degree of invasiveness

It is a non-invasive parameter. *T*_c-toe_ is affected by hypothermia and room temperature.

### Skin mottling

#### Background

Skin mottling is defined as “patchy skin discoloration.” Skin mottling usually manifests around the knees and might extend to other sites of peripheral circulation such as fingers and ears [33]. Skin mottling is a result of heterogeneous small vessel vasoconstriction [38]. Skin mottling is an easily assessed sign of peripheral hypoperfusion [38].

#### Selectivity and sensitivity in clinical settings

A six-grade scoring system (ranging from 0 to 5) has been introduced by Ait-Oufella and colleagues depending on the extent of skin mottling around the knee [39]. Skin mottling score (SMS) ranges from 0: no mottling, 1: a coin sized mottling area at the knee center, 2: a mottling area that does not extend above the upper margin of knee cap, 3: a mottling area localized to the lower thigh, 4: a mottling area reaching the upper thigh below the groin fold, to 5: extensive mottling reaching above the groin fold [39]. SMS could be subjective estimate of the severity of peripheral hypoperfusion [39]. Higher SMS (scoring 4 to 5) was associated with poor patient outcome in general ICU population [40] and in patients with septic shock [41]. A good correlation was reported between changes in both mottling score and skin perfusion (assessed by laser Doppler) during resuscitation of septic shock patients [42]. Prolonged skin mottling more than 6 h was associated with ICU mortality independently from severity scoring systems [40].

#### Usefulness in other settings

There is no available evidence.

#### Limitations and degree of invasiveness

It is a non-invasive parameter. It has a limited value in burns, amputations, and in dark skin.

### Capillary refill time (CRT)

#### Background

CRT is defined as “the time needed for skin’s color to return to baseline on a finger’s tip after application of blanching pressure” [33]. CRT can be clinically measured over the fingertip [35, 43] or over the knee area [43]. CRT is a measure of peripheral capillary blood flow [33].

#### Selectivity and sensitivity in clinical settings

CRT showed a good correlation with urinary output and serum lactate levels [43]. Prolonged CRT has been associated with poor outcomes in septic shock patients [43] and in non-selected critically ill patients [34] with a cutoff value that ranged between 2.5 and 5 s. Normalization of CRT was associated with improved survival rate in septic shock patients [44].

#### Usefulness in other settings

Prolonged CRT has also been associated with poor outcomes in patients following abdominal surgery [35].

#### Limitations and degree of invasiveness

It is a non-invasive parameter. Index CRT has the disadvantage of inter-rater variability. Knee CRT has a limited value in dark skin.

### Peripheral perfusion index (PPI)

#### Background

PPI represents “the ratio between the pulsatile and non-pulsatile component of the light reaching the pulse oximeter” [45]. As the pulsatile (arterial) flow is the only portion affected with vasoconstriction and vasodilatation, PPI has been considered as a numerical non-invasive measure for peripheral perfusion. PPI decreases in states of hypoperfusion due to decreased pulsatile component with a constant non-pulsatile component of blood flow [45].

#### Selectivity and sensitivity in clinical settings

The change in PPI reflects the change in other measures of hypoperfusion such as temperature gradients [45], lactate, and P (v-a) CO_2_ [46]. In critically ill patients, PPI less than 1.4 is a marker of hypoperfusion [45]; also, PPI less than 0.6 is an independent factor for 30-day mortality [46]. In septic shock patients, PPI less than 0.3 predicted vasopressor therapy [47] and below 0.2 predicted mortality [48].

#### Usefulness in other settings

Persistent decrease in PPI is associated with poor outcome after therapeutic hypothermia for out-of-hospital arrest [36] and in major abdominal surgery patients [35].

#### Limitations and degree of invasiveness

PPI is a non-invasive measure; however, it needs a special pulse oximeter. PPI is characterized by high skewness [45] and high inter-individual variability [49]; thus, it is more useful in trend monitoring for follow-up rather than being used as a single measure [49].

### Tissue oxygen saturation (StO_2_)

#### Background

StO_2_ is measured using near-infrared spectroscopy (NIRS) [6]. Measurement by StO_2_ is based on the difference in light absorption between oxy- and deoxyhemoglobins [50]. StO_2_ represents the hemoglobin oxygen saturation in the tissues [6]. The normal value for StO_2_ is 87% which represents the sum of arterial, venous, and capillary blood oxygen saturation [51]. The most common sites for measurement of StO_2_ are thenar and frontal. StO_2_ decreases in states of hypoperfusion and hypoxia [50].

#### Selectivity and sensitivity in clinical settings

Thenar StO_2_ decreases in situations of hypoperfusion according to shock severity [51, 52]. In trauma patients, thenar StO_2_ could discriminate patients in severe shock [51]. Low thenar StO_2_ during initial resuscitation of multiple trauma patients correlate with future development of multiple organ dysfunction [53].

#### Usefulness in other settings

StO_2_ could predict the need of blood transfusion in trauma patients [54]. In cardiac surgery, cerebral oxygenation below 50% predicted poor postoperative cognitive dysfunction [55]; moreover, cerebral oxygenation showed good correlation with jugular venous saturation [56].

#### Limitations and degree of invasiveness

StO_2_ is a non-invasive parameter; however, it has some limitations: firstly, StO_2_ showed high variability among healthy volunteers, patients with mild and moderate shock; thus, its value is restricted to patients with severe shock [51]; secondly, this high variability makes StO_2_ a useful parameter for trend monitoring rather than a single measure value [49]. Thirdly, StO_2_ is of low value in patients with septic shock [6, 57].

### Continuous transcutaneous oxygen measurement

#### Background

Subcutaneous partial oxygen pressure (Ptco_2_) was previously measured using invasive subcutaneous probes [58]; however, newer technology facilitated Ptco_2_ measurement using non-invasive transcutaneous probes [59, 60]. Ptco_2_ has been more popular in neonates than adults [61]. The limited use of Ptco_2_ in adults is because of the lack of agreement between its value and arterial oxygen tension due to the thicker epidermis in adults compared to neonates [61].

#### Selectivity and sensitivity in clinical settings

Despite its low accuracy in adults, Ptco_2_ has been reported as a useful predictor for outcomes in critically ill emergency patients [59, 60]. A newer parameter, the oxygen challenge test (OCT), was reported as a tool for early diagnosis of poor peripheral perfusion [48, 62, 63]. OCT the Ptco_2_ response to increasing Fio_2_ to 1 for a 5-min duration. The Ptco_2_ increased with increasing FiO_2_ in non-shocked patients, whereas Ptco_2_ poorly responded to increasing FiO_2_ in shocked patients. An increase of 21 mmHg in Ptco_2_ after OCT was associated with better patient outcome [62]. Poor response to OCT was a predictor of mortality when reported before [62, 63] or after [48] resuscitation. In a randomized controlled trial, Hu et al. [64] investigated the use of OCT as a resuscitation target in patients with severe sepsis; they reported that a response of 25 mmHg or more to OCT would improve the patient outcome.

#### Usefulness in other settings

There is no available evidence.

#### Limitations and degree of invasiveness

Although it is a non-invasive measure, Ptco_2_ has some limitations. Ptco_2_ needs special monitor and electrodes. Moreover, there is no definitive cutoff value. The value of OCT is restricted to intubated patients only.

### Sublingual microcirculation

#### Background

Visualization of sublingual microcirculation using handheld microscopes has recently gained widespread attention. Direct visualization of microcirculation focuses on vascular density, flow, and proportion of perfused vessels [65]. Assessment of microcirculation has been a research tool not applicable in the clinical bedside assessment. With the introduction of more advanced microscopes (CytoCam), bedside real-time assessment of microcirculation has been available with adequate agreement with the conventional image analysis [66].

In a cross-sectional multicenter observational study, Lima et al. [67] investigated the inter-rater reliability for 45 physicians and 16 nurses in subjective assessment of sublingual microcirculation; participants were asked to categorize the videos into good, bad, and very bad microcirculation. Results of the aforementioned study showed good agreement between the participant assessment and the conventional analysis [67]. A recent protocol for real-time assessment of microcirculation was recently introduced by Naumann et al. [68]. Point of care microcirculation (POEM) is a 5-point scoring system that could be used for assessment of flow and heterogeneity with accepted inter-rater agreement [68].

#### Selectivity and sensitivity in clinical settings

It is still under research.

#### Usefulness in other settings

There is no available evidence.

#### Limitations and degree of invasiveness

Assessment of sublingual microcirculation is a non-invasive procedure. However, it has the disadvantage of the need of an expensive device.

## Perfusion-guided resuscitation

Although normalization of central indices of tissue perfusion (such as lactate and ScvO_2_) has been an essential target of resuscitation of patients with septic shock [69], studies validating the value of targeting an improved peripheral perfusion markers are sparse. In an observational study, Hernandez and colleagues reported that early recovery of peripheral perfusion indices (such CRT, *T*_c-toe_, and P (v-a) CO_2_) is a marker of successful resuscitation of septic shock patients. In an animal study, Van Genderen et al. [70] reported a relationship between central and peripheral markers of perfusion during resuscitation of experimental shock; however, the rate of normalization of perfusion markers was dependent on the underlying type of shock. A randomized controlled trial (RCT) investigated the perfusion-based approach during resuscitation of 30 septic shock patients was conducted by the same group of authors [71]. The aforementioned study reported that the perfusion-based approach resulted in lower fluid replacement, less organ dysfunction, and shorter length of stay [71]. Larger RCTs are recommended to validate the perfusion-based approach and to set more clear steps of resuscitation.

## Conclusions

Monitoring of tissue perfusion includes biomarkers of global tissue perfusion, measures for assessment of perfusion in non-vital organs, and direct visualization of sublingual microcirculation.

Three main clinical implications for perfusion indices were reported. First, the presence of poor tissue perfusion in a shocked patient is usually associated with worse outcome. Second, persistently impaired perfusion despite adequate resuscitation is associated with worse outcome. Third, normalization of some perfusion indices (lactate and central venous oxygen saturation) has become one of the resuscitation targets in patients with septic shock.

Direct visualization of sublingual circulation represents a promising tool for evaluation of peripheral perfusion. Simple and user-friendly protocols are being introduced to facilitate the use of sublingual circulation as a bedside tool for assessment of shocked patients.

Although the collective evidence shows the clear relation between impaired peripheral perfusion and mortality, the use of different perfusion indices as a resuscitation target was not adequately investigated. Most of the data concerning the perfusion indices are extracted from observational trials with a few number of randomized controlled trials.

## Abbreviations

CRT: Capillary refill time
DO_2_: Oxygen delivery
ICU: Intensive care unit
LVS: Left ventricular strain
NIRS: Near-infrared spectroscopy
OCT: Oxygen challenge test
P (v-a) CO_2_: Central-venous-arterial CO_2_ gap
POEM: Point of care microcirculation
PPI: Peripheral perfusion index
Ptco_2_: Subcutaneous partial oxygen pressure
RCT: Randomized controlled trial
ScvO_2_: Central venous oxygen saturation
SMS: Skin mottling score
StO_2_: Tissue oxygen saturation
SvO_2_: Mixed venous oxygen saturation
*T*_c-toe_: Central-to-toe temperature
*T*_skin-diff_: The temperature gradient between fingertip and forearm
